# Decline of heterozygosity in a large but isolated population: a 45-year examination of moose genetic diversity on Isle Royale

**DOI:** 10.7717/peerj.3584

**Published:** 2017-07-17

**Authors:** Renae L. Sattler, Janna R. Willoughby, Bradley J. Swanson

**Affiliations:** 1 Department of Biology, Central Michigan University, Mount Pleasant, MI, United States of America; 2 Alaska SeaLife Center, Seward, AK, United States of America; 3 Department of Biological Sciences, Purdue University, West Lafayette, IN, United States of America

**Keywords:** Alces alces, Genetic drift, Inbreeding, Dispersal, Microsatellites, Population genetics, mtDNA

## Abstract

Wildlife conservation and management approaches typically focus on demographic measurements to assess population viability over both short and long periods. However, genetic diversity is an important predictor of long term population vitality. We investigated the pattern of change in genetic diversity in a large and likely isolated moose (*Alces alces*) population on Isle Royale (Lake Superior) from 1960–2005. We characterized samples, partitioned into five different 5-year periods, using nine microsatellite loci and a portion of the mtDNA control region. We also simulated the moose population to generate a theoretical backdrop of genetic diversity change. In the empirical data, we found that the number of alleles was consistently low and that observed heterozygosity notably declined from 1960 to 2005 (*p* = 0.08, *R*^2^ = 0.70). Furthermore, inbreeding coefficients approximately doubled from 0.08 in 1960–65 to 0.16 in 2000–05. Finally, we found that the empirical rate of observed heterozygosity decline was faster than the rate of observed heterozygosity loss in our simulations. Combined, these data suggest that genetic drift and inbreeding occurred in the Isle Royale moose populations over the study period, leading to significant losses in heterozygosity. Although inbreeding can be mitigated by migration, we found no evidence to support the occurrence of recent migrants into the population using analysis of our mtDNA haplotypes nor microsatellite data. Therefore, the Isle Royale moose population illustrates that even large populations are subjected to inbreeding in the absence of migration.

## Introduction

Though wildlife conservation and management approaches typically focus on demographic measurements to assess population viability over both short and long periods, increased attention is being placed on including an assessment of genetic diversity as a measurement of population health and vitality. Genetic diversity is lost through stochastic genetic drift at a rate inversely related to population size. Reduced genetic diversity has been related to a reduction in fitness associated with increased inbreeding (Cassinello, 2005; Marshall & Spalton, 2006), which can initiate an extinction vortex (Gilpin & Soulé, 1986). In an extinction vortex, a decrease in population size increases inbreeding, resulting in a decrease in reproductive success, further reducing population size, creating a negative feedback loop that successively increases the probability of extinction each generation (Frankham, 1995; Lacy, 1997; Dennis, 2002). This cycle is particularly problematic in isolated populations, as without input of novel genetic diversity into the population, the population does not typically recover (Frankham, 2015; Akesson et al., 2016).

Increasing gene flow through dispersal can alleviate the negative impacts associated with inbreeding by reducing the effect of intra-specific competition (Myers & Krebs, 1971; Nee & May, 1992; Hanski & Selonen, 2008) and facilitating demographic and genetic rescue (Brown & Kodric-Brown, 1977). Under a genetic rescue scenario, inbreeding depression may be alleviated after only a single generation, as the divergent alleles carried by the immigrant offset the deleterious but typically recessive alleles found in the source population (e.g., Vila, 2003; Bouzat et al., 2009), with the benefits persisting for multiple generations (Frankham, 2016). Accordingly, high levels of dispersal are not required to alleviate inbreeding depression as the immigration of even a single individual into a highly inbred declining population can rapidly spread new alleles and lead to rapid population growth (Vila, 2003; Heber et al., 2013).

**Figure 1:**
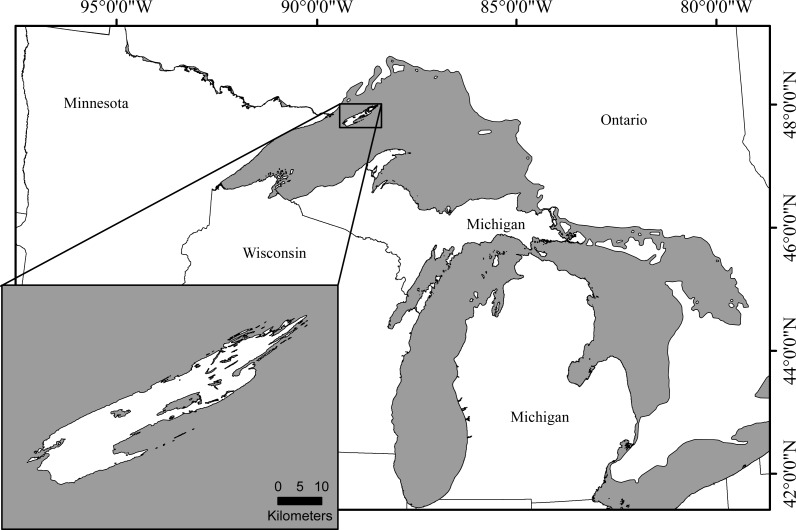
Isle Royale National Park, Michigan. The island is approximately 80 km long and 12 km wide and is located in the northwestern portion of Lake Superior. At its shortest Euclidean distance, the island is 24 km off the southern coast of Canada.

The likelihood of extinction increases with inbreeding severity and decreasing population size (Bijlsma, Bundgaard & Boerema, 2000; Willi & Hoffmann, 2009). Furthermore, inbreeding depression increases with stressful conditions (Bijlsma, Bundgaard & Van Putten, 1999; Joubert & Bijlsma, 2010; Haanes et al., 2013) and populations experiencing limited gene flow have a lower adaptive potential than non-fragmented populations (Bakker et al., 2010). While many lab-based studies have provided valuable information, finding naturally isolated wild populations with sufficient demographic and genetic data to evaluate change in genetic variation over time is difficult. Isle Royale is a small isolated island, approximately 80 km long, 12 km wide, and located, at its shortest Euclidean distance, 24 km off the southern coast of Canada (Fig. 1) providing an opportunity to study genetic change under naturally isolated conditions. While Lake Superior has made colonization of the island difficult for many mammalian species, it is currently home to nearly twenty mammal species, including the North American moose, *Alces alces*, and gray wolves, *Canis lupus*.

The Isle Royale moose population was founded in the early 1900s with the first census reporting approximately 200 moose in 1915 (Mech, 1966). How moose came to Isle Royale remains largely unknown. Initially, it was proposed moose walked across the ice (Murie, 1934), but this idea was quickly questioned, citing moose’s aversion to ice and swimming to be a more probable explanation (Mech, 1966; Haanes et al., 2013). In 1998, anecdotal evidence surfaced suggesting that in 1907 11–13 moose were captured in northeastern Minnesota and transported to the island (Peterson, 1998). While genetic analyses provided some support for the Minnesota origin theory (Hundertmark, 1998), there has still been considerable speculation surrounding the source population and the mechanism by which moose founded the population on Isle Royale. Regardless, the idea that the moose population is isolated from surrounding mainland populations has been supported by previous genetic analyses (Wilson et al., 2003).

**Figure 2:**
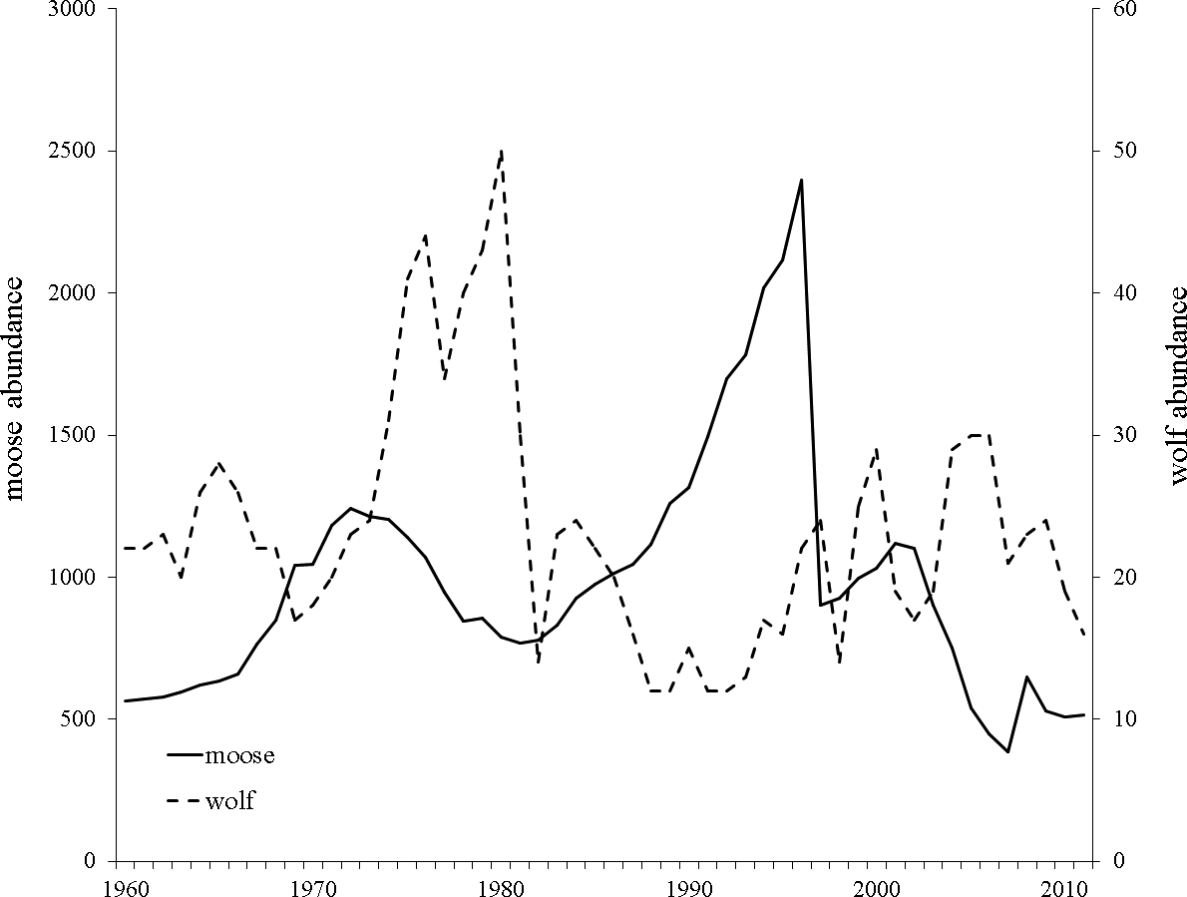
Yearly Isle Royale moose (*Alces alces*) and wolf (*Canis lupus*) population census size since 1959. Moose population size is presented with a solid black line and wolf population size with a dashed black line. Data obtained from http://www.isleroyaleowolf.org (Vucetich & Peterson, 2012).

Wolves colonized Isle Royale in the late 1940’s creating an almost single predator-single prey system in which moose constitute 90% of wolf diet (Peterson & Page, 1988). Yearly moose and wolf censuses have been conducted since 1959 and show moose density has ranged from 0.71–4.41 moose/km^2^ and averages approximately 1.80 moose/km^2^. Large fluctuations in moose and wolf population sizes have been documented and include a 42% decline in moose population size between 1972 and 1981, and a more pronounced, rapid population decline between 1996–1997 that reduced the moose population by 80% (1,922 individuals; Post et al., 2002; Fig. 2). Necropsies determined starvation as the primary cause of death, a result of a record setting winter with respect to snow depth and temperature (Peterson, 1997).

The Isle Royale moose population has been isolated since the early 1900s and 60 years of consecutive data collection provides the rare opportunity to assess genetic variation over multiple generations in a naturally isolated population. Using the Isle Royale moose population as a model system, we evaluated three main objectives. First, we quantified changes in genetic diversity in the isolated population of moose on Isle Royale over a 45-year period. We hypothesized that, due to the isolating nature of Lake Superior, genetic drift and inbreeding should reduce genetic diversity over time. Secondly, we investigated the occurrence of recent immigration of moose onto Isle Royale. We hypothesized that moose immigration had not occurred due to moose aversion to ice and the long-distance swim required in cold water. Thirdly, we utilized the highly-detailed moose demographic history to simulate the change in diversity over time to compare and assess potential causes for differences in simulated verses empirically derived estimates of change. We hypothesized that, because the island population was likely isolated, genetic drift would be a strong force acting to remove genetic diversity over time.

## Materials and Methods

Isle Royale researchers have collected moose carcasses from Isle Royale National Park since 1958. Approximate age, likely cause of death, and sex are known for >4,500 carcasses. We haphazardly selected 55 moose born within each of five sampling periods: 1960–1965, 1970–1975, 1980–1985, 1990–1995, and 2000–2005 for analysis. Sampling periods were chosen to minimize overlap of generations and because they included the extremes of the moose population fluctuations (Fig. 2).

DNA was obtained from the pulp cavity of teeth by dissecting the teeth into <1.5 cm sections of the root cavity using a Dremel 300 (Robert Bosch Tool Corporation, Racine, WI, USA) as well as any tissue attached to the sides of the teeth. The DNA was extracted using Qiagen DNeasy kits following published protocol (Qiagen Valencia, CA, USA), quantified with a NanoDrop Spectrophotometer ND-1000 (Thermo Scientific, Wilmington, DE, USA), and diluted to a concentration of 15 ng/µl.

All moose samples were amplified at nine microsatellite loci, using primers BM757, BM4513, BM848 (Bishop et al., 1994), MAF70 (Buchanan & Crawford, 1992), MAF46 (Swarbrick et al., 1992), McM58 (Hulme et al., 1994), RT5, RT9, RT30 (Wilson et al., 1997) via polymerase chain reaction (PCR) using an Eppendorf Mastercycler Gradient (Eppendorf, Westbury, NY, USA). PCRs were completed using Qiagen Multiplex Kit (Qiagen, Valencia, CA, USA). Primers MAF70, McM58 and BM848 had an optimized annealing temperature of 60 °C and comprised one multiplex grouping. Primers BM757, RT9 and BM4513 comprised a second multiplex grouping with an annealing temperature of 57 °C. The final multiplex grouping of primers MAF46, RT5 and RT30 had an optimal annealing temperature of 58 °C. We used 50 µl reactions containing 50–100 ng of DNA, 0.2 µM of each forward and reverse primer, 0.5x Q-Solution and 1X Qiagen Multiplex PCR Master Mix with a 3 mM concentration of MgCl_2_ and 15 µl RNase-free water. PCR conditions consisted of 15 min at 95 °C followed by 30–35 cycles of 94 °C for 30 s, 57 °–60 °C for 90 s, and 60 s at 72 °C with a final extension period of 10 min at 72 °C. Amplified DNA was analyzed using an ABI Prism 310 Genetic Analyzer (Applied Biosystems, Foster City, CA, USA). Allele size was determined using GENESCAN v. 3.1.2 and Genotyper v. 2.0 with TAMRA 500 base-pair size standard (Applied Biosystems, Foster City, CA, USA). Samples that were not successfully genotyped using multiplex kits were reamplified in 10 µl single primer PCR’s containing 50–100 ng of template DNA, 125 µM dNTP’s, 0.16 µM each of forward and reverse primer, 1x Buffer, and 0.375 units Hotmaster Taq Polymerase (5 PRIME, Gaithersburg, MD, USA). PCR cycles were performed as follows: denaturing of DNA for 2 min at 94 °C, followed by 30–35 cycles at 94 °C for 45 s for denaturing primer specific annealing at 57°−60 °C for 45 s and a final extension at 65 °C for 10 min (Broders et al., 1999). A sample negative was included in all PCR plates for quality control.

Prior to statistical analysis, we used MICRO-CHECKER to test microsatellite genotypes for the presence of null alleles, repeat motif consistency, scoring errors and allelic dropout (Van Oosterhout et al., 2004). We used GENEPOP on the web (Raymond & Rousset, 1995; Rousset, 2008) to test all microsatellite loci for deviations from Hardy Weinberg Equilibrium using probability testing with a Bonferroni corrected alpha (α = 0.0055; Rice, 1989). Additionally, all pairs of loci in each population were tested for linkage disequilibrium using log-likelihood ratio statistics with a Bonferroni corrected alpha (α = 0.0014). Markov chain parameters were set to 1,000 dememorizations, 100 batches with 1,000 iterations per batch.

To better understand how genetic diversity changed over time, we measured estimates of heterozygosity and inbreeding at each time period and determined the pattern of change observed over the study period. First, using GENALEX 6.3 (Peakall & Smouse, 2005), we estimated the number of alleles and observed heterozygosity for each sample period. We also estimated inbreeding coefficients (F_IS_) for each locus and across each sample period using the R Demerelate package (Kraemer & Gerlach, 2017; R Core Team, 2016) following Weir & Cockerham (1984). Finally, we used simple linear regressions to determine if our estimates of genetic diversity (number of alleles, observed heterozygosity, F_IS_) changed over time (five, 5-year time periods beginning in 1960–65 and ending in 2000–05). In all three linear regressions, significant models were denoted by a slope that was significantly different from zero.

Population substructuring was assessed within and across sample periods using non-spatially explicit program, STRUCTURE (Pritchard, Stephens & Donnelly, 2000), and a spatially explicit program, BAPS (Corander et al., 2008). Given the recently highlighted complications of estimating population structure using microsatellites (Putman & Carbone, 2014), we required both model-based clustering analyses to indicate population substructure to have confidence in its presence. We ran five independent STRUCTURE runs using *K* = 1 − 9 for each sample period and for all sample periods combined, assuming correlated allele frequencies and admixture. Structure results were visualized using Structure Harvester (Earl & vonHoldt, 2012). Similarly, BAPS was run for five replicates of *K* = 1 − 9 for each sample period and the combined time period dataset, using the clustering of individuals option and no spatial prior. For both analyses, we used a burn-in of 100,000 steps and 100,000 replicates (Falush, Stephens & Pritchard, 2003), which allowed convergence.

We looked for evidence of migration onto Isle Royale using two genetic markers: (1) mtDNA and (2) microsatellite genotypes. First, 134 samples partitioned across our sample periods were selected for mitochondrial sequencing. DNA was extracted and run on a 1% agarose gel prior to PCR to check for DNA degradation. Gels were stained with SybrGold (Molecular Probes, Eugene, OR, USA) and examined on a UV transilluminator. The portion of DNA closest to the well was excised and cleaned using Qiagen MinElute Kits (Qiagen Valencia, CA, USA). DNA was amplified at the left hyper-variable domain of the control region via polymerase chain reaction (PCR) using an Eppendorf Mastercycler Gradient (Eppendorf, Westbury, New York) and primers LGL283 and ISM015 (Hundertmark et al., 2002). We used 20 µl PCR reactions containing 25–100 ng of DNA, 125 µM dNTP’s, 0.2 uM of forward and reverse primer, 1 × Buffer containing 1.5 mM MgCl_2_, 1 × Flexi Buffer, 1unit of GoTaq Polymerase (Promega Corporation, Madison, WI, USA) and 6.45 µl ultrapure water. Polymerase chain reaction conditions consisted of 2 min at 94 °C followed by 35 cycles of 94 °C for 45 s, 50 °C for 45 s, and 45 s at 72 °C with a final extension period of 10 min at 72 °C. Resulting sequences were aligned to known moose haplotypes from multiple populations across North America and Europe using BLASTn from the National Center for Biotechnology Information (http://blast.ncbi.nlm.nih.gov/Blast.cgi, last accessed 05/2017) to ensure our sequences were moose. All 134 sequences were verified as moose, and were subsequently aligned using the program MACVECTOR 7.2.3 (Cary, NC, USA http://www.macvector.com). Prior to analyses, the first and last 30 base pairs of each sequence were removed to reduce false identification of mutations. We used the ClustalW Alignment tool in MACVECTOR for multiple alignments to identify mutations and calculate the number of sequences per sample period. ClustalW parameters were set to a 10.0 open gap penalty, 5.0 extended gap penalty, 40% delay divergent, and weighted transitions.

In addition to examining mtDNA for a signal of immigration, we also examined our microsatellite data for evidence of recent migration onto Isle Royale. Specifically, we used an assignment test in GENECLASS2 (Piry et al., 2004), which is able to identify the probability an individual within a dataset originated from the genotyped population(s), even if the source population was not sampled or genotyped. We analyzed each sample period using the Baysian framework (Rannala & Mountain, 1997), with 10,000 iterations and the default threshold *p* value (type I error) of 0.01. Assignment probability was calculated using the Monte Carlo resampling method of Paetkau et al. (2004) which limits simulated populations to the same sample size as reference populations thereby more accurately reflecting sampling variance.

Finally, we simulated the loss of microsatellite genetic diversity in the Isle Royale moose population to provide a theoretical baseline for comparison against the observed change in diversity. The R simulations began by simulating 564 individuals, equal to the population size of moose on Isle Royale in 1960, and by assigning 2 alleles per locus for each individual, using the empirical allele frequencies from the 1960–1965 dataset. We used this initial population as a starting point, and allowed the microsatellite allele frequencies to evolve in future years by assigning newly created (*i.e.,* born) individuals alleles that were randomly selected from the parental genotypes of that individual. To simulate reproduction, we selected breeders by first limiting female breeding age to between two and 15 years (Schwartz & Hundertmark, 1993) and male breeding age between five and 12 years (Mysterud, Solberg & Yoccoz, 2005) and then randomly selecting from the remaining pools. We incorporated mutation into the models, assuming a mutation rate of 10^−4^ (Schlötterer, 2000; Bulut et al., 2009), by allowing step-wise mutations during allele assignment. For all individuals, we assigned male/female with a probability of 0.5 for both sexes and assigned age (1–15 years) with equal probability in the first simulated year and incrementally increased age of individuals in subsequent years. Finally, we limited population growth by adhering to the known Isle Royale moose population census data and removing individuals over age 15 from the simulated population. In years of population increase, we increased the population from the current number to the next year census size with new offspring. In years of population decline, we randomly selected individuals from the population for input into the next simulated year. The simulation was run for 100 iterations to quantify the variability associated with our estimates. We calculated the mean observed heterozygosity and number of alleles in each year across all replicates.

## Results

We successfully genotyped 251 moose samples with 41–54 samples per locus, in each sample period (See Supplemental Information 3). Results from MICRO-CHECKER did not indicate the presence of null alleles, scoring errors or allelic dropout. All nine microsatellite loci in all sample periods were in Hardy Weinberg Equilibrium (HWE; all *P* > 0.0055) and no loci were linked (all *P* > 0.0014) after Bonferroni corrections.

**Figure 3:**
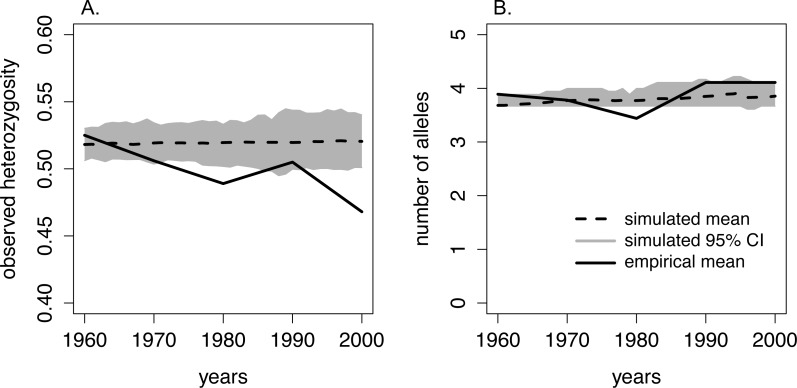
Comparison of heterozygosity and the number of alleles between the empirical data from moose on Isle Royale and simulated Isle Royale moose populations. We conducted simulations, beginning with allele frequencies observed in 1960, and compared the simulated genetic diversity estimates to the empirical estimates gathered from a suite of microsatellites. Empirical genetic diversity estimates are indicated by the solid line and reflect the mean (A) heterozygosity and (B) number of alleles for each of our five sampling periods. The simulated estimates of heterozygosity and number of alleles are show for each year, beginning in 1960 and ending in 2000. Simulated means are noted with the dashed line, and the 95% CI around the means are depicted with the grey band.

We found that the number of alleles per locus from 1960–2005 ranged from 1–7, and the average number of alleles per sample period ranged from 3.7–4.1 (Table 1, Fig. 3). The number of alleles present in the population was not significantly related to sampling period (Table 2). However, observed heterozygosity displayed a marginally significant, negative relationship over time: observed heterozygosity was 0.53 in 1960–65 and 0.47 in 2000–05 (Table 1, Table 2, Fig. 3). Furthermore, F_IS_ values approximately doubled from 0.08 in 1960–65 to 0.16 in 2000–05. However, this increase was not statistically significant over the study period (Table 1, Table 2).

**Table 1:** The number of alleles (N_a_), observed heterozygosity (H_o_), expected heterozygosity (H_e_) and inbreeding coefficient (F_IS_) of the Isle Royale moose population per locus from 1960 to 2005. The sample size (*n*) in each time period is denoted in parenthesis.

	1960–65 (*n* = 51)				1970–75 (*n* = 55)				1980–85 (*n* = 50)				1990–95 (*n* = 50)				2000–05 (*n* = 45)			
	N_a_	H_o_	H_e_	F_IS_	N_a_	H_o_	H_e_	F_IS_	N_a_	H_o_	H_e_	F_IS_	N_a_	H_o_	H_e_	F_IS_	N_a_	H_o_	H_e_	F_IS_
MAF46	4	0.55	0.53	0.05	3	0.60	0.54	−0.11	2	0.60	0.50	−0.18	3	0.61	0.51	−0.19	2	0.33	0.50	0.34
MAF70	4	0.73	0.66	0.00	4	0.71	0.67	−0.04	3	0.66	0.62	−0.08	6	0.60	0.63	0.08	5	0.71	0.67	−0.04
BM757	5	0.42	0.45	0.14	5	0.72	0.70	−0.02	5	0.56	0.62	0.11	5	0.60	0.67	0.12	6	0.64	0.68	0.07
BM848	4	0.57	0.61	0.17	4	0.46	0.56	0.18	4	0.55	0.57	0.03	4	0.44	0.51	0.17	4	0.39	0.54	0.28
BM4513	3	0.71	0.48	−0.38	3	0.44	0.42	−0.04	5	0.67	0.56	−0.20	6	0.67	0.59	−0.13	5	0.54	0.53	0.01
McM58	5	0.83	0.68	−0.10	5	0.81	0.64	−0.27	5	0.61	0.66	0.08	5	0.65	0.59	−0.08	5	0.73	0.65	−0.12
RT5	3	0.06	0.06	−0.53	3	0.07	0.06	−0.02	1	0.00	0.00	NA	1	0.00	0.00	NA	3	0.05	0.05	−0.01
RT9	4	0.51	0.58	0.15	5	0.60	0.60	0.02	4	0.55	0.56	0.04	4	0.71	0.67	−0.05	4	0.64	0.56	−0.13
RT30	3	0.35	0.33	0.20	2	0.15	0.12	−0.07	2	0.20	0.25	0.20	3	0.27	0.24	−0.10	3	0.20	0.18	−0.08
**Average**	**3.88**	**0.53**	**0.49**	**0.08**	**3.78**	**0.51**	**0.48**	**0.13**	**3.44**	**0.49**	**0.48**	**0.18**	**4.11**	**0.51**	**0.49**	**0.13**	**4.11**	**0.47**	**0.48**	**0.16**

**Table 2:** Linear regression results comparing the number of alleles (N_a_), observed heterozygosity (H_o_), and inbreeding coefficient (F_IS_) over time in the Isle Royale moose population from 1960–2005. The study period was comprised of five, 5-year time periods beginning in 1960–65 and ending in 2000–05.

	*F*-statistic	*P*-value	*R*^2^	Coefficient	Coefficient estimate	Standard error
N_a_	0.76	0.45	0.20	Intercept	3.627	0.300
				Slope	0.079	0.090
H_o_	6.75	0.08	0.70	Intercept	0.538	0.015
				Slope	−0.012	0.005
F_IS_	2.40	0.22	0.44	Intercept	0.094	0.033
				Slope	0.016	0.010

We found no evidence of population substructure within or across sample periods using STRUCTURE or BAPS. Using the combined dataset with all sample periods, STRUCTURE and BAPS identified *K* = 1 as the most likely number of populations (probability = 1, BAPS; Supplemental Information 1). Although the mean penalized likelihood score from STRUCTURE suggested that some sample periods had a secondary peak and, therefore, substructure (*K* = 5 − 7) when the sample periods were run individually, the probability of assignment for individuals within each dataset was approximately equal to 1/*K*. This pattern suggests that the secondary peaks may be artifacts of fitting a small sample size (*n* = 47 − 52) into a relatively large number of populations (*K* = 5 − 9). Collectively, we interpret these trends as support for a single population that lacked substructure in Isle Royale moose.

In addition to examining our populations for evidence of substructuring, we also looked for a genetic indicator of recent migration of moose onto Isle Royale. Using the sequences generated for the left hypervariable region of the control region, we found only one mitochondrial haplotype in the 134 samples sequenced from Isle Royale. The total sequence length ranged from 390–555 nucleotides per individual (see Supplemental Information 3). This haplotype corresponded to Hundertmark et al. (2003) haplotype L, which was only found in the moose population identified as Central North America (*n* = 23) and included samples from north-eastern and north-central Minnesota, southwest Ontario, Isle Royale, northeast North Dakota and the Lake Winnipeg area of Manitoba (Table 3). We also examined our microsatellite data for signals of recent migration into the Isle Royale moose population. We found no evidence of migrants in our dataset within any of our five time periods (*i.e.,* no moose had a significant likelihood of not originating from the Isle Royale moose population), using the assignment tests in GENECLASS2. Because GENECLASS2 will identify migration events even when the source population is unsampled, we interpret these data as evidence supporting the notion that the moose population on Isle Royale was isolated.

**Table 3:** Frequency of Central North American moose (*Alces alces*) haplotypes calculated from Hundertmark et al. (2003). The haplotype found in the Isle Royale moose population corresponds to haplotype L.

Central North America Haplotype	Frequency of Haplotype in Central North America
J	26%
K	4.4%
L	39%
M	4.4%
N	26%

When comparing observed heterozygosity and the number of alleles between the simulated and empirical populations, the declining trend in empirical heterozygosity diverged from the constant heterozygosity level over time demonstrated in our simulated population. While the empirical heterozygosity levels observed in 1960–65, 1970–75, and 1990–95 fell within our simulated 95% CI of heterozygosity, empirical heterozygosity in 1980–85 and 2000–2005 was significantly lower than the predicted heterozygosity by the simulated population (Fig. 3). Additionally, in the 1980–85 sample period the number of alleles was lower than estimated by our simulated population (Fig. 3).

## Discussion

Our main objectives were to quantify the changes in genetic variation over forty-five years in a naturally isolated moose population, investigate the occurrence of recent migrants into the population, to use simulations to establish a baseline of heterozygosity loss for comparison to empirical values, and to determine the factors that may have influenced the observed changes. We found decreasing heterozygosity accompanied by increasing rates of inbreeding likely associated with a small founding population size and subsequent genetic drift. Although inbreeding can be mitigated by migration, we found no support for the occurrence of immigration into the population.

Our genetic diversity estimates were similar to those found in the Isle Royale moose population by Wilson et al. (2003; H_e_ = 0.48, *n* = 15), and to two introduced and subsequently isolated populations in South Central Alaska with similar founding size (Kalgin Island H_o_ = 0.47, *n* = 19; Berners Bay H_o_ = 0.53, *n* = 8; Hundertmark, 2009). When compared to neighboring Canadian mainland moose populations, heterozygosity levels on Isle Royale were notably lower and F_IS_ values higher (Wilson et al., 2003). The significant decrease in moose heterozygosity and increase in F_IS_ values over our 45-year study period suggests that the moose population on Isle Royale were impacted by genetic drift and increased inbreeding, which is often associated with isolated populations.

While moose immigration onto Isle Royale is unlikely, its occurrence would have confounded our assessment of factors influencing changes in genetic diversity over the course of the study. Therefore, we first looked for immigration to the island by comparing the frequency of mitochondrial haplotypes on Isle Royale to the haplotype frequencies reported in six North American moose populations (Hundertmark et al., 2003). The population identified as the Central North American population, which included samples from Manitoba, Ontario, North Dakota, Minnesota and Michigan, had 5 haplotypes with frequencies ranging from 4.4 to 39% (Table 3). Using the 134 sequenced samples spanning our study period, we should have been able to detect haplotypes on Isle Royale that occurred with a frequency of at least 0.7%. However, we found only a single haplotype (L) on the island. Of the five haplotypes identified in the Central North American moose population (Table 3), the haplotype on Isle Royale corresponded to moose from northeast Minnesota and North Dakota (Hundertmark, 1998; Hundertmark et al., 2003; K Hundertmark, pers. comm., 2016). Mainland Canadian moose populations experience moderate to high connectivity (Wilson et al., 2003) and if Lake Superior does not impede immigration then we should have found a haplotype frequency distribution similar to that of the mainland, or at least the presence of additional haplotypes. While haplotype L had the highest frequency in the Central North American moose population (39%), a random moose immigrating to the island from the adjacent mainland populations had a 61% probability of carrying any haplotype other than haplotype L (Table 3). Furthermore, we also examined our microsatellite data, which is transmitted by both males and females, for evidence of recent migrants into the population. However, these data did not yield any significant signals of immigration. Therefore, our data do not appear to support the occurrence of recent moose immigration onto the island.

While young or adult moose of either sex may disperse from their natal areas (Labonte et al., 1998), the majority of dispersers are subadult males (Garner & Porter, 1990; Hundertmark, 2007; Singh et al., 2012), which would have been undetected in our mtDNA analyses but not our microsatellite analyses. While moose are capable of long distance dispersal events in the absence of geographical barriers (1,000 km, Niedzialkowska et al., 2016), more often dispersal distances are described as typically short (Feldhamer, Thompson & Chapman, 2003). The mean dispersal distance in a sample of 18 juvenile interior Alaskan moose was 3 km (Gasaway et al., 1985) and, in a population of moose inhabiting southern Sweden, migration distances ranged from 4.4–5.7 km (Singh et al., 2012). At its shortest straight-line distance, Isle Royale is 24 km from southern coast of Ontario, and there are no other islands between Isle Royale and any surrounding mainland that would offer rest or a visual target. Furthermore, we provide additional empirical evidence that the haplotype frequencies present on Isle Royale are dissimilar to adjacent populations (Hundertmark et al., 2003) and thus natural colonization seems unlikely. These findings also corroborate the previous genetic (Hundertmark, 1998) and anecdotal report (Peterson, 1998) that the Isle Royale moose population was likely founded by human intervention. Cumulatively, these data suggest that dispersal to the island is, at best, rare and that the Isle Royale moose population is effectively isolated from mainland moose populations.

The comparison of our empirical and simulated heterozygosity (observed) yielded a notable divergence in overall trends over the 45-year period covered in our study. First, empirical heterozygosity displayed an overall declining trend whereas simulated populations maintained heterozygosity over this time period. Second, empirical heterozygosity levels in the 1990–95 sample period deviated from the overall declining trend, and this corresponded to an increase in population size on the island. Our simulations were constrained by the Isle Royale moose census sizes and, theoretically, experienced the same degree of genetic drift as the Isle Royale population. The overall quicker reduction of empirical heterozygosity compared to simulated heterozygosity suggests that the Isle Royale moose population was experiencing an ecological pressure that was unaccounted for in our simulations. Although this deviation could be caused by sub-structuring of the moose population, we were unable to detect multiple populations using either BAPS or STRUCTURE. Alternatively, these deviations may have been caused by fluctuations in the Isle Royale effective population size not captured by our model. For example, deviations in our assumed generation time and lifespan would have resulted in a better retention of rare alleles, slowing the rate of loss of heterozygosity (Willoughby et al., 2013; Kimble, Rhodes Jr & Williams, 2014). However, we chose conservative parameter values for our simulations that should not have completely erased the signal of heterozygosity loss, particularly over only five generations.

One possible mechanism driving the observed decrease in heterozygosity is the incidence of inbreeding occurring in the moose population on the island. Although not statistically increasing over the course of our study, the increase in F_IS_ may have been biologically significant and caused the loss in heterozygosity we observed over consecutive generations (Fig. 3). This is supported by the empirical estimate of heterozygosity in 1990-1995, which was an outlier in the declining heterozygosity trend over the study period and had a smaller inbreeding coefficient compared to the two adjacent generations (Fig. 3; Table 1). The increase in heterozygosity and decrease in inbreeding observed in the 1990–95 data coincided with a sharp increase in moose population size (Fig. 2). Although the precise reason for the temporary demographic release of the moose population is unknown, shifting ecological pressure may have been responsible. Beginning in the 1980s, canine-parvovirus decimated the Isle Royale wolf population and the population was never able to recover to its previous census size (Peterson et al., 1998). The drastic reduction in the number of wolves on the island led to a shift in the ecological pressure controlling the moose population from a predator-controlled (top down) system to a mixture of climate and food-availability (bottom up) factors driving population dynamics (Peterson et al., 1998; Vucetich & Peterson, 2012). As a result of the decrease in wolf predation pressure, a period of significant moose population growth occurred; the Isle Royale moose population reached a 50-year peak size of approximately 2,500 individuals over the early to mid 1990s (Fig. 2). This period of rapid population growth was accompanied by a reduction in inbreeding (Table 1), likely due to increased mate availability. Therefore, the increasing moose population size in the 1990s was likely responsible for the reduced rate of inbreeding accumulation, suggesting that the previous population size, although large, was still not sufficient to prevent increasing levels of inbreeding on the island. Thus, our data support the notion that inbreeding is a significant force that acts to degrade heterozygosity over time, even in large and robust populations, in the face of isolation.

##  Supplemental Information

10.7717/peerj.3584/supp-1 Supplemental Information 1 Structure penalized likelihood plot for all samples from all time periods Circles represent the mean likelihood and bars show the 95% confidence interval for each estimate.

10.7717/peerj.3584/supp-2 Supplemental Information 2 Simulation code

10.7717/peerj.3584/supp-3 Supplemental Information 3 Isle Royale moose microsatellite genotypes and mtDNA data
