# Supplementary material for: Decline of heterozygosity in a large but isolated population: a 45-year examination of moose genetic diversity on Isle Royale
Source: PeerJ. 2017 Jul 17;5:e3584. doi: 10.7717/peerj.3584 (PMC5516768; doi:10.7717/peerj.3584)
Supplement: Supplemental Information 1 — Circles represent the mean likelihood and bars show the 95% confidence interval for each estimate. [file peerj-05-3584-s001.docx]

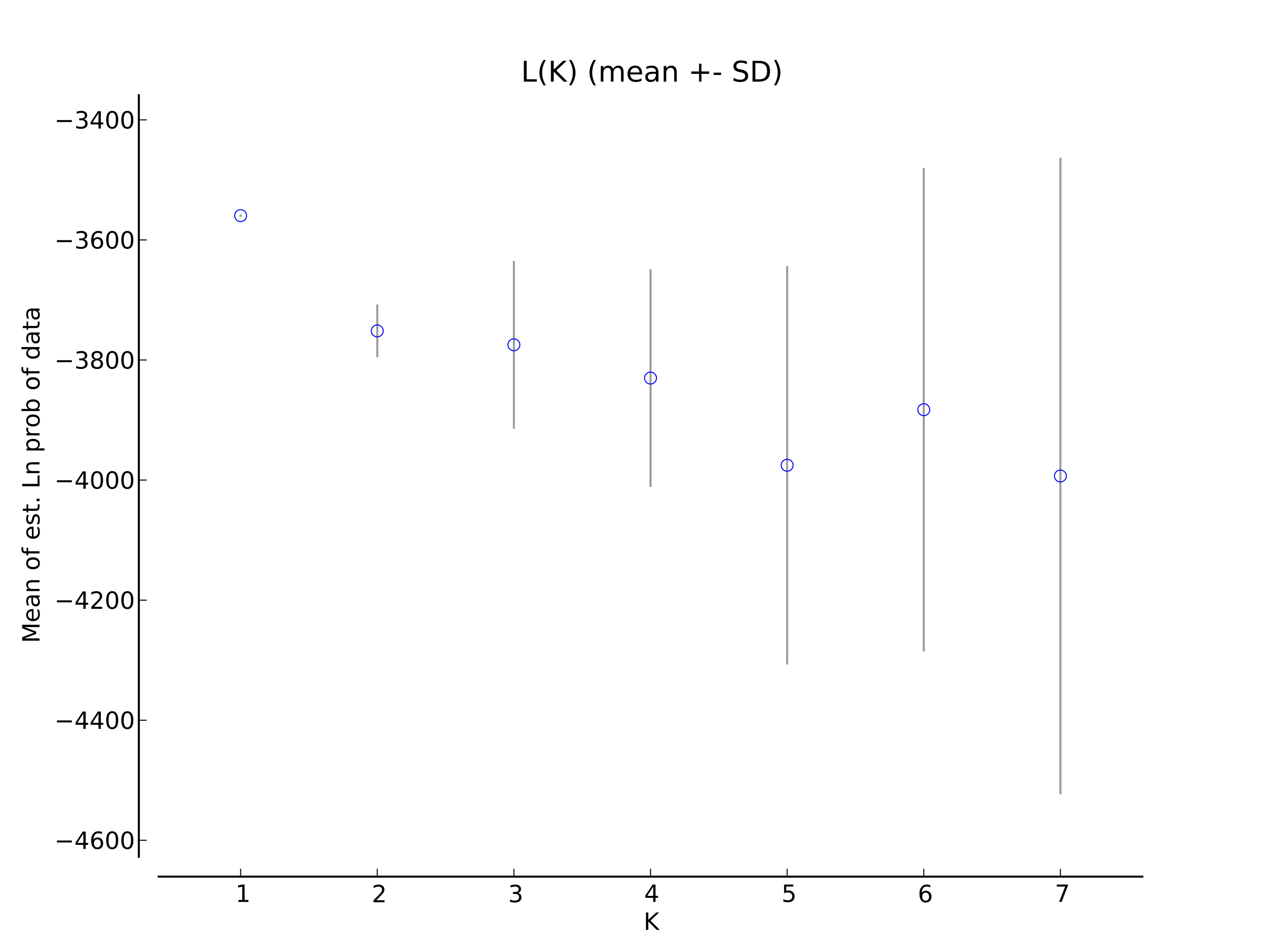


SI Fig. 1. Structure penalized likelihood plot for all samples from all time periods. Circles represent the mean likelihood and bars show the 95% confidence interval for each estimate.
